# Evolutionary game theory for physical and biological scientists. I. Training and validating population dynamics equations

**DOI:** 10.1098/rsfs.2014.0037

**Published:** 2014-08-06

**Authors:** David Liao, Thea D. Tlsty

**Affiliations:** Department of Pathology, University of California San Francisco, San Francisco, CA 94143, USA

**Keywords:** game theory, coevolution, modelling, cancer, population dynamics, therapeutic scheduling

## Abstract

Failure to understand evolutionary dynamics has been hypothesized as limiting our ability to control biological systems. An increasing awareness of similarities between macroscopic ecosystems and cellular tissues has inspired optimism that game theory will provide insights into the progression and control of cancer. To realize this potential, the ability to compare game theoretic models and experimental measurements of population dynamics should be broadly disseminated. In this tutorial, we present an analysis method that can be used to train parameters in game theoretic dynamics equations, used to validate the resulting equations, and used to make predictions to challenge these equations and to design treatment strategies. The data analysis techniques in this tutorial are adapted from the analysis of reaction kinetics using the method of initial rates taught in undergraduate general chemistry courses. Reliance on computer programming is avoided to encourage the adoption of these methods as routine bench activities.

## 1. Introduction

In this paper, we will describe a method of data analysis that can be used by physical and biological scientists to analyse population dynamics using game theoretic replicator equations. This method is based on the method of initial rates taught in undergraduate chemistry courses.

**Figure 1.**
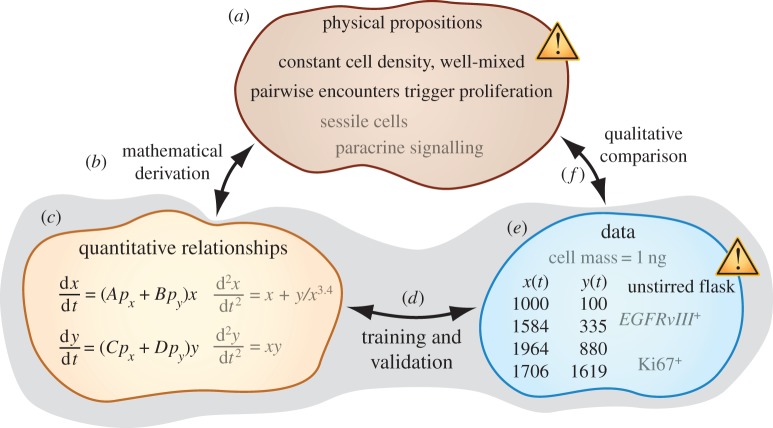
Overview of physical sciences modelling. To determine whether we have developed a set of physical propositions that accurately describe experimental data, we can (*f*) directly compare (*a*) propositions and (*e*) experimental knowledge using qualitative language. For example, we find that the model in this cartoon needs to be revised because it relies on a proposition of thorough mixture that disagrees with an experimental procedure involving an unstirred flask. Complicated model propositions and abundant data can often render such word-model comparisons impractical. Indirect comparison can be achieved using the proxy of fitting equations. In an accompanying manuscript [1], we describe how (*a*) physical models are used in (*b*) mathematical derivations to obtain (*c*) equations. The focus of this paper, shaded in grey, is to illustrate a method for (*d*) comparing fitting equations with (*e*) experimental data. We refer to this comparison as ‘training and validation’. (Online version in colour.)

In §2, we provide background to describe the interdisciplinary need that we hope that this tutorial will help to address. The tutorial is organized with the help of figure 1. For the purposes of this discussion, we define mathematical modelling as the development of a consistent set of physical propositions (assumptions), quantitative relationships (equations, qualitative shapes of function plots, etc.) and observations (data). In §3, we show how equations and data can be compared using parameter training and model validation. In an experimental report in this Theme Issue, Wu *et al.* [2] apply aspects of this style of approach to study the dynamics of multiple myeloma (MM) and stromal cell populations interacting in microfabricated structures. In an accompanying manuscript [1], we show how fitting equations and physical propositions can be associated using mathematical derivations. One benefit of performing such derivations is to make clear that multiple sets of propositions can be consistent with a set of equations, so that no particular set of propositions should necessarily be accepted on faith. We conclude in §4 by illustrating a potential clinical impact of the data analysis method described in §3.

## 2. Need for a tutorial in game theoretic analysis of cell population dynamics

Game theory is sometimes regarded as a promising candidate for elucidating features of cancer (and other areas of biology) in ways that provide both basic understanding and insights into therapeutic treatment. At the same time, the application of game theory can be challenging. This tutorial is presented both to help interdisciplinary investigators to realize potential insights and to help investigators to avoid common pitfalls in mathematical modelling.

At interfaces connecting the physical and biological sciences, a perspective is emerging that severe limitations in our ability to understand and control cancer are results of naive applications of reductionist reasoning to ‘complex’ systems [3,4]. In systems biology, reductionism and complex systems thinking are commonly portrayed by comparing those molecular biologists who focus intently on a small number of genes or on a small number of pathways, on the one hand, with, on the other hand, quantitative biologists who investigate the generic, common properties (e.g. topologies and motifs) of gene-regulatory networks and who also try to understand why these properties might demonstrate universality [5]. This kind of distinction can also be made at other scales, for example when comparing cell-centric thinking with integrative perspectives that highlight interactions between cells and the cellular and non-cellular components of their surrounding tissue environments.

A growing number of physical scientists and biologists view game theory as a potentially fruitful framework for describing the evolution of interacting cell populations in a way that will provide understanding of cancer progression and therapeutic control [6]. In an accompanying manuscript, we clarify why this form of modelling is called ‘evolutionary game theory’ (EGT) [1]. For the purposes of this paper, we use EGT simply to refer to models of cell interactions in which net expansion rates for cell subpopulations depend on the frequency with which different cell types are represented in the overall population. In this sense, the phrase ‘frequency-dependent models’ would serve our purposes equally well. Applications of EGT and associated dynamical models have produced ecological and evolutionary insights in a variety of biological systems including communities of budding yeast cells that share metabolic public goods [7–9] and populations of *Escherichia coli* that use colicins to antagonize each other in a cyclic, or ‘rock–paper–scissors’, fashion both *in vitro* [10] and *in vivo* [11]. Of particular relevance to this Theme Issue, mathematical models of cell–cell interactions have also been applied to investigate the dynamics of various cancer systems [12–15]. Two of these examples illustrate an archetypically EGT style of analysis. Dingli *et al.* [14] use continuous-time replicator equations to study the dynamics and equilibria of mixed populations of MM, osteoclast (OC) and osteoblast cells. Their analysis provides an insight that direct attempts to eradicate the MM cell population are likely to lead to relapse of disease because partial cytoreduction does not alter the long-term equilibrium composition of the three-population system, but, instead, simply ‘reset[s] the clock’ in an inevitable approach towards MM and OC dominance. As a second approach, the authors recommend ‘changing the rules of engagement between different cell types … literally *changing the dynamics*, enabling normal cells to out-compete the malignant clone, consequently leading to its evolutionary extinction’ [14, p. 7]. In a second example, Basanta & Anderson [6] use a four-population model to gain insights into the cell–cell interactions leading to the promotion of the invasive (motile) phenotype in secondary glioblastoma multiforme. In addition to finding that suppressing angiogenic benefit can promote the invasive phenotype, the authors also predict that eventual dominance of the invasive phenotype might be preceded by transient oscillations in population composition.

Together, these examples showcase EGT and related mathematical models of cell–cell interactions as promising strategies for gaining insights into the behaviour of complex ecologies that have not yet been fully elucidated using reductionist approaches. Unfortunately, interdisciplinary fields present special risks for misunderstanding and confusion when fitting equations to data. In a recent example drawn from the interface of psychology and nonlinear dynamics, incorrect data analysis methods were used to support a claim that ‘flourishing’ personalities could be distinguished using a ‘critical positivity ratio’ [16]. The Lorenz equations were misapplied to describe the dynamics of speech acts in business teams. These equations simplistically describe atmospheric convection and have no clear relevance to psychology. Additionally, data were not plotted alongside theoretical curves for direct comparison. Rather than being trained by data, some model parameters were assigned values historically used by mathematicians merely to make unrelated teaching illustrations. Owing to these and additional weaknesses, the claim to have found a critical positivity ratio was criticized for being ‘entirely unfounded’ [16, p. 1].

Taken together, the examples in this section suggest that it is important to disseminate skill in the application of EGT modelling to data analysis, not only because such analysis might provide basic science and clinical insights, but also because it can be challenging to correctly apply mathematical modelling in an interdisciplinary setting.

## 3. Phase portrait comparison of differential equations and population dynamics

In this section, we show how an ecology consisting of two populations can be analysed using EGT replicator equations. In §3.1, we show how parameters can be trained from data, and in §3.2, we show how differential equations containing the trained parameters can be validated by comparing them with additional data not initially used for training. If differential equations are validated, they can be used to make predictions about additional experiments, as described in §3.3.

**Figure 2.**
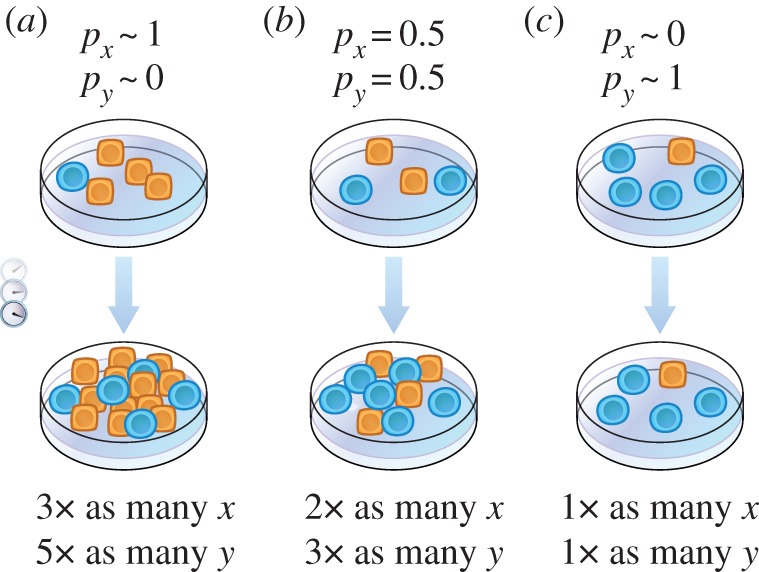
Population dynamics that depend linearly on demographic composition. Three co-cultures of cells of type *x* (square, yellow) and cells of type *y* (round, blue) are prepared with different initial population compositions: (*a*) almost exclusively composed of cell type *x*, (*b*) 50/50 mixture of both cell types and (*c*) almost exclusively composed of cell type *y*. The factors by which each subpopulation then expands are also varied. (Online version in colour.)

In the cartoon in figure 2, the numbers of cells in two populations cultured together are monitored over time. We label these populations *x* and *y* and represent them using square, yellow cells and round, blue cells, respectively. The proportion of cells that are of type *x* is *p_x_* := *x*/(*x* + *y*), and the proportion of cells that are of type *y* is *p_y_* := *y*/(*x* + *y*). Three different initial compositions are shown. In figure 2*a*, the population is rich in square, yellow cells. In other words, *p_x_* is nearly unity, and *p_y_* is nearly zero. In figure 2*b*, the proportions are initially equal, i.e. *p_x_* = *p_y_* = 0.5. Figure 2*c* starts with an initial composition that is rich in the round, blue cells. Here, *p_x_* is nearly zero, whereas *p_y_* is nearly unity. The fraction by which each population increases over time varies with population composition. For example, in scenario (*a*) population *x* increases to a factor of 3 times its original value, whereas in scenario (*b*) population *x* increases only to a factor of 2 times its initial value. In scenario (*c*) population *x* does not expand. In this simplistic cartoon, the population expansion factors 3, 2 and 1 and the corresponding values of the initial fractions of cells of type *x*, *p_x_* ∼ 1, 0.5 and ∼ 0, respectively, are consistent with a linear relationship between expansion factor and initial population fraction. A set of differential equations approximately containing this linear relationship3.1
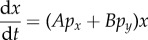
3.2
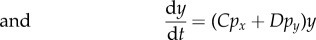
is written by setting the instantaneous rate of change of population *x* over time *t* equal to a product between a rate coefficient (‘fitness’) *f_x_* = *Ap_x_* + *Bp_y_* and the population size *x*, as well as by setting the instantaneous rate of change of population *y* over time equal to a product between a rate coefficient *f_y_* = *Cp_x_* + *Dp_y_* and the population size *y*. Having specified the differential equations to be compared with data, we show in §3.1 how the parameters *A*, *B*, *C* and *D* can be determined from time-course measurements of cellular populations.

Before continuing, we clarify a point of possible confusion. Equations (3.1) and (3.2) describe the dynamics of absolute population *numbers*, but ecological and evolutionary biologists commonly write down governing equations for the dynamics of population *fractions* (examples presented in the electronic supplementary material). This is done either in a form that can be directly derived from equations (3.1) and (3.2) [17] or in a form using an alternative measure of time [18]. Analysing the dynamics of population fractions requires fewer equations and parameters. For example, the population fraction dynamics corresponding to equations (3.1) and (3.2) can be written as a single equation involving only two parameters (or three parameters using the alternative measure of time), rather than the four independent parameters *A*, *B*, *C* and *D*. However, this simplicity does not come without cost. An analysis at the level of population fractions might predict a decrease in the fraction of a relatively aggressive cell type, but if the overall population increases over the same time period, the absolute size of the aggressive subpopulation might also have increased, leading to disease progression. Owing to the possibility of neglecting clinically relevant information when analysing the dynamics of population fractions alone, we deliberately use equations describing the dynamics of population numbers in this tutorial. We will refer to equations (3.1) and (3.2) as replicator dynamics equations even though this phrase often refers to equations describing population fraction dynamics.

### 3.1. Training

**Figure 3.**
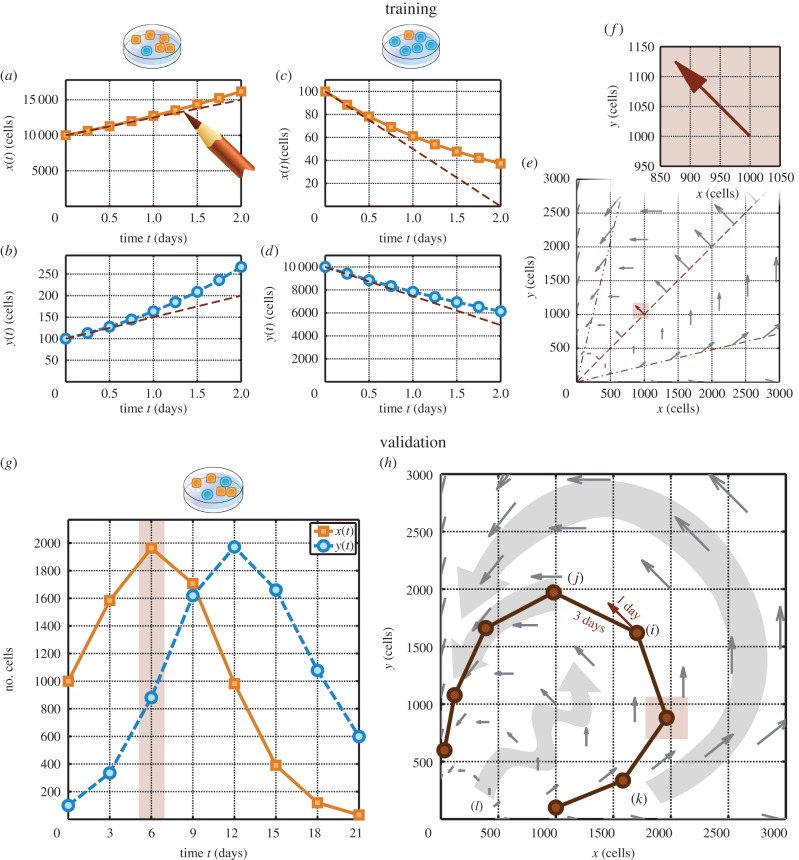
Method for training and validation of population dynamics equations for an ecology consisting of two populations. The numbers of cells of type *x* (*a*) and of type *y* (*b*) are monitored for a co-culture initially rich in cells of type *x*. The numbers of cells of type *x* (*c*) and of type *y* (*d*) are monitored for a different co-culture initially rich in cells of type *y*. The slopes in (*a*)–(*d*) are used to specify the velocity field in (*e*), with magnified inset in (*f*), as described in the main text. A separate dataset (*g*) is compared with the velocity field in (*h*) to check for agreement in magnitude and direction. Population compositions (*j*), (*k*) and (*l*) are examples of initial co-culture compositions that could be tested in additional experiments. (Online version in colour.)

In this section, we apply a version of the ‘method of initial rates’ [19,20], familiar from undergraduate chemistry courses, to train the parameters in equations (3.1) and (3.2). The main idea of this method is that individual equation parameters can be isolated by considering how population sizes vary while one subpopulation dominates. Figure 3 shows three hypothetical co-culture datasets, (*a*,*b*), (*c*,*d*) and (*g*). Panels (*a*,*b*) form one dataset corresponding to a co-culture that is initially highly enriched in cells of type *x*, with *x*(0) = 10 000 cells and *y*(0) = 100 cells at initial time *t* = 0. For these panels, *p_x_* is nearly unity and *p_y_* is nearly zero, so equation (3.1) becomes3.3
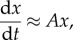
a simple proportionality between the instantaneous rate of change of population *x* and the population size *x* itself. This implies that3.4
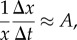
which means that the coefficient *A* is approximated by the product of the reciprocal of the population size *x* and the initial rate at which *x* changes. In figure 3*a*, population *x* increases with curvature. The slope of the line tangent to the earliest data points covers a rise of Δ*x* = +5000 cells over the course of Δ*t* = 2 days. Substituting these values, along with the initial population size of 10 000 cells, into equation (3.4), we estimate3.5
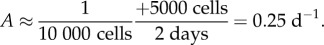
We have used the initial slope of the *x* versus *t* graph for a co-culture initially rich in cells of type *x* to estimate coefficient *A*. The mathematically inclined reader might note that equation (3.4) is equivalent to stating that the rate of change of the natural logarithm of *x* approximates *A*. Using a plot of ln(*x*) versus *t* to estimate *A* can provide a longer duration of time over which the initial slope at time *t* = 0 is reasonably approximated by the average slope over a finite interval of time, but, for the purposes of this tutorial, it suffices to perform an analysis of population size versus time rather than an analysis of the natural logarithm of population size versus time.

Applying arguments analogous to those we used to obtain equations (3.3)–(3.5) allows us to use the slope of population *y* versus time in (*b*) and equation (3.2) to estimate that3.6
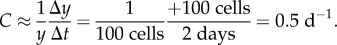


To estimate the remaining parameters, we consider a co-culture initially rich in cells of type *y*. Setting *p_x_* nearly equal to zero and *p_y_* nearly equal to unity in equations (3.1) and (3.2) now allows us to use the plot of population *x* versus *t* in panel (*c*) and the plot of population *y* versus *t* in panel (*d*) to estimate that3.7
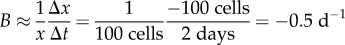
and that3.8
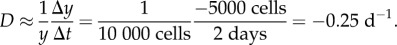


Now that we have determined the parameter values by analysing the dynamics of co-cultures with initially *x*- or *y*-rich population compositions, we fill in a ‘velocity plot’ to graphically represent our trained differential equations. To understand this procedure, we consider a hypothetical population composed initially of 1000 cells of type *x* and 1000 cells of type *y*, located at the tail of the arrow in figure 3*f*. We estimate the change in population *x* accrued over a short interval of time3.9
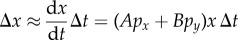
by multiplying the instantaneous rate of change of *x* specified according to equation (3.1) by the duration of the time interval Δ*t*. Substituting parameter values *A* = 0.25 d^–1^ and *B* = −0.5 d^–1^, along with *p_x_* = *p_y_* = 0.5 (because both subpopulations are equal), *x* = 1000 cells, and Δ*t* = 1 day, we obtain3.10
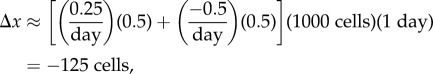
a decrease that brings the original population size of 1000 cells down to 1000 – 125 cells = 875 cells. Analogous reasoning applied to equation (3.2) allows us to estimate that population *y* increases by 125 cells during the same day, yielding a final population of 1000 + 125 cells = 1125 cells. The position (875 cells, 1125 cells) is indicated by the head of the arrow in panel (*f*). This arrow represents population change over the course of a day. The remaining arrows in (*e*) are obtained in the same way as we have highlighted using panel (*f*). Panel (*e*) is sometimes referred to as a ‘phase portrait’ or a ‘velocity plot’.

A brute-force calculation of each arrow in (*e*) would be tedious. To improve efficiency, we take advantage of a geometric property of equations (3.1) and (3.2). The dash-dotted line is shallow, the dash-double-dotted line is steep and the dashed line has intermediate slope. Each of these lines is an example of a set of positions in the *x*–*y* plane sharing a common population composition. For example, all points on the dashed line have a population composition of *p_x_* = *p_y_* = 0.5 because at all of these points the *x* and *y* population sizes equal each other. Repeating the calculation of the change in population *x* demonstrated in equation (3.10) at other values of *x* along this line only requires replacing the factor of 1000 cells with other values of *x*. All other factors serve as a constant coefficient. Thus, along the dashed line, the values of Δ*x* are simply proportional to the initial values of *x* used to locate the tails of the illustrated arrows. Following similar reasoning, values of Δ*y* are also proportional to the initial values of *y*. Taken together, these observations lead to the property that the arrows originating from the dashed line grow longer in proportion to distance from the origin and share the same direction. The same conclusions can be drawn for the arrows originating from the dash-double-dotted and dash-dotted lines, respectively. Sketching only a handful of arrows thus allows us to quickly fill in a dense velocity plot if the initially drawn arrows are judiciously chosen so that they do not all lie on a shared line containing the origin.

### 3.2. Validation

Now that we have graphically represented changes in population size that would be consistent with equations (3.1) and (3.2), we compare the phase portrait in (*e*) with an additional dataset in panel (*g*) that explores population compositions not yet explored by the data in panels (*a*)–(*d*). At the highlighted time point (*t* = 6 days), the co-culture contains about 2000 cells of type *x* and 900 cells of type *y*. This corresponds to the highlighted point in (*h*). Plotting pairs of *x*- and *y*-population values at other times in the same way fills out a loop that circulates in the counterclockwise direction. This example is constructed so that equations (3.1) and (3.2) validate. To see how the loop is consistent with the background field of arrows, we assess agreement in terms of both magnitude and direction. For example, consider data point (*i*) and the arrow highlighted nearby. The direction of this arrow and the direction of the line segment joining point (*i*) to point (*j*) roughly agree (some discrepancy is possible given that the time points are separated by a coarsely granular step of 3 days). Additionally, the length of the arrow and the length of the line segment also agree. The line segment is approximately three times as long as the arrow, a ratio consistent with the ratio of durations of time that the line segment and arrow are supposed to represent. By observing qualitative agreement between the dataset in (*g*) and the velocity field that was specified using the other datasets in (*a*)–(*d*), we have completed a preliminary, qualitative validation of equations (3.1) and (3.2).

### 3.3. Prediction

We referred to the procedure in §3.2 as a *preliminary* validation. The phase portrait in (*h*) contains a variety of arrows describing population dynamics anticipated for a wide array of starting population sizes and compositions. The datapoints in (*g*) that we used to validate the phase portrait probe only a small set of these arrows. To challenge the fitting equations further, we plan additional experiments; three examples are shown in (*j*), (*k*) and (*l*). We would perform an experiment with the initial population composition at point (*j*) to determine whether ensuing population dynamics would follow the grey arc predicted to advance from (*j*) in a counterclockwise fashion. If the dynamics were different, we could speculate that the dynamics advancing from an initial population composition depended on the history of the culture preceding that ‘initial’ composition. In other words, we would consider a possible ‘memory effect’ not accounted for in equations (3.1) and (3.2). In experiment (*k*), the initial sizes both of population *x* and of population *y* are larger than the corresponding initial values from the dataset in (*g*). If the counterclockwise loop originating from (*k*) were observed, the phase portrait would be further validated. The phase portrait predicts that a small counterclockwise loop would be obtained by preparing a co-culture with smaller values for both *x* and *y*, for example at point (*l*). If the ensuing dynamics traced out a wiggle, the phase portrait would be invalidated, and we would conclude that equations (3.1) and (3.2) failed to describe the experimental system.

While we have used a simple two-population cartoon to illustrate procedures for training, validation and the planning of additional experiments, the same approach and analogous equations can be applied to systems containing more than two populations, as described in the electronic supplementary material.

## 4. Discussion

In this tutorial, we have shown how replicator dynamics equations can be trained and validated using population sizes measured in co-culture over time. We close by discussing potential clinical implications.

### 4.1. Clinical implications

The purpose of modelling is not merely to obtain a consistent set of propositions, equations and data. Particularly in cancer research, we wish to obtain strategies for optimizing clinical treatment and insights into basic biology.

**Figure 4.**
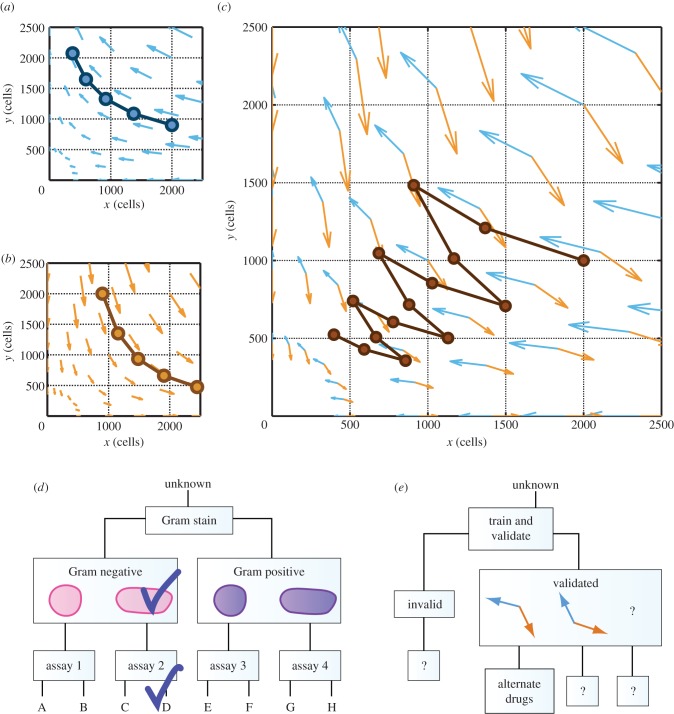
Proposed clinical impact of phase portrait analysis for therapeutic scheduling. (*a*) Hypothetical treatment condition leads to unbounded expansion of population *y*. (*b*) Alternative treatment condition leads to unbounded expansion of population *x*. (*c*) Alternating between the two conditions generates a ‘tacking’ trajectory that gradually decreases both populations *x* and *y*. (*d*) Simplified pathogen-identification ‘rule out’ chart. (*e*) Hypothesis that the geometric insights described in (*a*)–(*c*) might eventually be incorporated into a physical sciences–oncology therapy planning chart. (Online version in colour.)

Dingli *et al.* [14] proposed ‘changing the dynamics’ as a therapeutic aim of applying EGT to cancer. Figure 4 shows an example of a potential strategy that the phase portrait analysis described in this tutorial can help us to hypothesize. Under one drug, a two-population system might be described using the velocity field in (*a*). When a different drug is applied, the dynamics of the population might be altered to conform to a different velocity field, as in (*b*). If either cell type is sufficient to cause disease, then neither treatment applied alone produces a desirable outcome. The treatment in (*a*) leads to unbounded expansion of population *y*, whereas the treatment in (*b*) leads to unbounded expansion of population *x*. However, a scheduled combination of the two drugs eventually reduces both populations. Panel (*c*) shows that the quivers from (*a*) and (*b*) form angles of less than 180° that approximately face the origin, (*x*, *y*) = (0, 0). This geometric property has the consequence that alternating between the two drug treatments in sequence will reduce both cell populations in a ‘tacking’ trajectory. If the angles in (*c*) opened away from the origin, then the alternating drug schedule would cause both populations to expand. This example illustrates one way that quantitative thinking can provide insights beyond those obtained through verbal reasoning alone. Because treatments (*a*) and (*b*) reduce populations *x* and *y*, respectively, an analysis based on word models would probably have identified a combination of treatments (*a*) and (*b*) as potentially beneficial in reducing both populations *x* and *y*. However, it would have been difficult to recognize the angle between the blue and orange quivers in (*c*), on the basis of word models alone, as a potentially critical predictor for the success of a combination schedule.

Comparing velocity fields corresponding to different drug treatment conditions can help in the identification of combination treatment schedules to achieve desired outcomes. Just as a clinician uses the visual appearance of a stained cell culture and a ‘rule out’ tree, as in (*d*), to identify a pathogen infecting a patient, oncologists working alongside physical scientists might one day be able to use the geometric properties of velocity fields and a physical sciences–oncology ‘rule out’ chart, as in (*e*), to determine which personalized drug schedules could be beneficial for a particular patient. Such a chart would realize a longstanding hope that understanding cell–cell interactions could point to improved strategies for drug combinations and time-sequenced scheduling in cancer therapy [21].

## Supplementary Material

Supplemental text and figures

## Data accessibility

Electronic supplemental materials, including slides for teaching and MatLab scripts for generating example plots, are available at http://qbio.lookatphysics.com/egt.php.
