# Supplementary material for: Evolutionary game theory for physical and biological scientists. I. Training and validating population dynamics equations
Source: Interface Focus. 2014 Aug 6;4(4):20140037. doi: 10.1098/rsfs.2014.0037 (PMC4071513; doi:10.1098/rsfs.2014.0037)
Supplement: Supplemental text and figures [file rsfs20140037supp1.pdf]

Supplemental materials
Evolutionary game theory for physical and biological scientists:
I. Training and validating population dynamics equations

David Liao and Thea D. Tlsty

Department of Pathology, University of California San Francisco, San Francisco, CA 94143

Sections and figures in this supplemental document are numbered to continue the numbering used in the main manuscript. Equations in the main manuscript are numbered from 3.1 through 3.10, and equations in this supplemental document are numbered S.11-S.28. Additional supplemental materials, including slides for teaching and MatLab scripts for generating example plots, will be available at http://qbio.lookatphysics.com/egt.php.

Contents

5. Curve-fitting for ternary and higher-order ecologies 2
5.1. Training 2
5.2. Validation 3
5.3. Prediction 4
6. Parameter values 5
6.1. Training and validation examples 5
6.2. Two-population examples 5
6.3. Three-population examples 7
Supplemental reference 8
Supplemental figures 9

5. Curve-fitting for ternary and higher-order ecologies

In this section, we show how the techniques demonstrated using the two-population system in section 3 of the main manuscript can be extended to a co-culture of three or more subpopulations. We slightly generalize equations 3.1 and 3.2 from the main manuscript by allowing the co-culture to contain an arbitrary number of subpopulations labeled *x*, *y*, . . . , *z*, where we take a notational shortcut by allowing letters of the alphabet, perhaps, to exist between *y* and *z* (nonetheless, to simplify figures, most of the discussion in this section uses the example of a 3-population system). We also rename the coefficients *A*, *B*, *C*, and *D* using indexed coefficients A_{xx}, A_{xy}, etc.

$$\frac{\mathrm{d}x}{\mathrm{d}t} = \left(A_{xx}p_x + A_{xy}p_y + \cdots + A_{xz}p_z\right)x \qquad \textbf{S.11}$$

$$\frac{\mathrm{d}y}{\mathrm{d}t} = \left(A_{yx}p_x + A_{yy}p_y + \cdots + A_{yz}p_z\right)y \qquad \textbf{S.12}$$

. . .

$$\frac{\mathrm{d}z}{\mathrm{d}t} = \left(A_{zx}p_x + A_{zy}p_y + \cdots + A_{zz}p_z\right)z \qquad \textbf{S.13}$$

Again, it is customary to refer to the rate coefficients as fitnesses. For example, the fitness of population *z* is labeled f_z and equals $A_{zx}p_x + A_{zy}p_y + \ldots + A_{zz}p_z$.

5.1. Training

Figure 5 illustrates that the parameter values in equations S.11-S.13 are trained using the same method that was used to train the parameter values in equations 3.1 and 3.2. Panel (*a*) shows that co-cultures of three cell types (*x*, *y*, and *z*) can be prepared so as to be almost purely enriched in cells of type *x*, almost purely enriched in cells of type *y*, or, as another alternative, almost purely enriched in cells of type *z*. The highlighted example is initially dominated by cells of type *x*. Setting $p_x \sim 1$ and the remaining population fractions p_y and p_z approximately equal to zero, equation S.12 becomes

$$\frac{\mathrm{d}y}{\mathrm{d}t} \approx A_{yx}y \qquad \textbf{S.14}$$

In the same way that was done using equation 3.6 in the main manuscript, the coefficient A_{yx} is estimated by measuring the slope of the line tangent to the plot of population *y* as a function of time *t*.

$$A_{yx} \approx \frac{1}{y}\frac{\Delta y}{\Delta t} = \frac{1}{50\,\text{cells}}\frac{+50\,\text{cells}}{4\,\text{days}} = 0.25/\,\text{day} \qquad \textbf{S.15}$$

The remaining parameters A_{xx} . . . A_{zz} can be obtained in a similar way, and their values for this example can be found in the following section of this supplemental document.

The final step in training equations S.11-S.13 is to draw a velocity field. Unfortunately, when describing the dynamics of more than two populations, it is difficult to draw arrows using coordinate axes directly analogous to the coordinate axes in Figure 3(*h*) from the main manuscript. Figure 5(*b*) illustrates an alternative that is especially useful for studying co-cultures of three populations. This triangular grid is called a simplex (ternary plot, de Finetti diagram [1]). Each point corresponds to a particular mixture of populations *x*, *y*, and *z*. The bottom-right corner is composed exclusively of cells of type *x*, the top corner is composed exclusively of cells of type *y*, and the bottom-left corner is composed purely of cells of type *z*. The highlighted arrow begins at the population composition $p_x = 0.4$, $p_y = 0.5$, and $p_z = 0.1$. To determine how these population fractions will change over time, we rewrite equations S.11-S.13 in terms of population fractions. Equation S.11 becomes

$$\frac{\mathrm{d}\,p_x}{\mathrm{d}t} = p_x\left[f_x - \left(f_x p_x + f_y p_y + \cdots + f_z p_z\right)\right] \qquad \textbf{S.16}$$

with analogous relationships for the remaining population fractions p_y . . . p_z. Substituting the trained values of the parameters A_{xx} . . . A_{zz} and the population fractions $p_x = 0.4$, $p_y = 0.5$, and $p_z = 0.1$ into equation S.16 allows us to estimate the change in population fraction p_x in the same way that we estimated the change in absolute population *x* in equations 3.9 and 3.10 in the main manuscript. Calculating changes in p_y and p_z then allows us to specify the population composition at the tip of the highlighted arrow in panel (*b*). In this diagram, each arrow represents a change in population composition corresponding to a time interval of 2 days.

5.2. Validation

Once we have sketched a velocity field on the simplex, we can compare it with the trajectory traced out by a dataset that was not initially used to train the parameters in equations S.11-S.13. In the simulation in (*c*), the population sizes at the highlighted time (t = 24 days) are x = 135 cells, y = 239 cells, and z = 32 cells, corresponding to the population fractions $p_x = 0.33$, $p_y = 0.59$, $p_z = 0.08$ and to the point highlighted in (*d*). Calculating the population fractions for the remaining time points in (*c*) fills out the rest of the

counterclockwise loop in (*d*). Just as in our two-population cartoon in Figure 3(*h*), the validation dataset in Figure 5(*c*) produces a trajectory that agrees with the trained velocity field in (*d*) in both direction and magnitude.

Before we consider panel (*d*) a successful validation, it is important to note that a simplex only displays population fractions, not absolute population sizes. This means that the dynamics of the absolute population sizes in (*c*) must be spot-checked to ensure that the rates of change of absolute population sizes are consistent with equations S.11-S.13. As an example, an approximate tangent line is drawn through the point y = 239 cells at t = 24 days. The slope of this dashed line is approximately +80 cells/6 days, or 13 cells/day. Substituting y = 239 cells, the population fractions $p_x = 0.33$, $p_y = 0.59$, and $p_z = 0.08$, along with the parameters $A_{xx} \ldots A_{zz}$, into equation S.12 predicts that population y achieves a rate of change of dy/dt = 15 cells/day, similar to the 13 cells/day measured. Analogous comparisons would need to be made at a variety of time points in (*c*) for multiple populations for us to be confident of the empirical accuracy of equations S.11-S.13. These spot-checks can be tedious. Unfortunately, these calculations become especially necessary when studying co-cultures of more than 3 populations. In such cases, validation is not convenient to perform on a simplex, which becomes a multidimensional structure that is difficult to draw on a sheet of paper.

5.3. Prediction

Now that we have performed a preliminary validation by using the velocity field in (*d*) and by measuring slope, as in (*c*), we can plan additional experiments to further test equations S.11-S.13. For example, we can ask whether preparing a co-culture initially rich in cells of type *z*, as in (*e*), would generate a rapid changeover toward a population rich in cells of type *x* (horizontal gray arrow). We can investigate whether the population composition at (*f*) is a steady-state. We can also investigate whether preparing a co-culture with initial population composition at (*g*) would recapitulate the counterclockwise loop already traced out by the dataset in (*c*) or, instead, produce qualitatively different dynamics like the oscillating gray path. Such an oscillation could suggest the presence of a memory effect for reasons similar to those discussed in subsection 3.3 in the main manuscript.

Because our examples in Figure 3 from the main manuscript and Figure 5 happen to involve velocity fields and trajectories that resemble counterclockwise loops, it is helpful to inspect the examples in Figure 6. These velocity fields demonstrate that equations S.11-S.13 can produce a variety of features including curves and lines that look nothing like closed cycles. Additionally, the following section shows that EGT-based models and equations can accommodate the dynamics of biological populations in which cell-cell interactions are not an important effect. Thus, even when a set of replicator equations is

validated, careful scrutiny is necessary to determine whether the data could be adequately described using equations based on a simpler model of interaction-independent growth.

6. Parameter values

Replication rate coefficients substituted into equations 3.1 and 3.2 from the main manuscript and equations S.11-S.13 from the supplemental materials are organized below according to the figures they were used to generate. In this section, we also argue that careful scrutiny is needed to look for situations in which simple models of interaction-independent growth would suffice to describe an experimental system.

6.1. Training and validation examples

Figure 3(*a*)-(*h*)

$$\begin{bmatrix} A & B \\ C & D \end{bmatrix} = \begin{bmatrix} 0.25/\text{day} & -0.5/\text{day} \\ 0.5/\text{day} & -0.25/\text{day} \end{bmatrix} \quad \textbf{S.17}$$

Figure 5(*a*)-(*d*)

$$\begin{bmatrix} A_{xx} & A_{xy} & A_{xz} \\ A_{yx} & A_{yy} & A_{yz} \\ A_{zx} & A_{zy} & A_{zz} \end{bmatrix} = \begin{bmatrix} 0.125/\text{day} & -0.25/\text{day} & 0.25/\text{day} \\ 0.25/\text{day} & 0 & -0.25/\text{day} \\ -0.25/\text{day} & 0.25/\text{day} & -0.25/\text{day} \end{bmatrix} \quad \textbf{S.18}$$

The parameter values in equations S.17 and S.18 happen to correspond to a prisoner's dilemma and a rough approximation of rock-paper-scissors, respectively. The names of these games are not important for the purposes of the tutorial.

6.2. Two-population examples

Figure 6(*a*)

$$\begin{bmatrix} A & B \\ C & D \end{bmatrix} = \begin{bmatrix} -0.15/\text{day} & -0.25/\text{day} \\ 0.15/\text{day} & 0.25/\text{day} \end{bmatrix} \quad \textbf{S.19}$$

Figure 6(*b*)

$$\begin{bmatrix} A & B \\ C & D \end{bmatrix} = \begin{bmatrix} -0.2/\text{day} & -0.2/\text{day} \\ 0.2/\text{day} & 0.2/\text{day} \end{bmatrix} \quad \textbf{S.20}$$

Figure 6(c)
$$\begin{bmatrix} A & B \\ C & D \end{bmatrix} = \begin{bmatrix} -0.3374/\text{day} & \text{-}0.0016/\text{day} \\ 0.1551/\text{day} & \text{-}0.3810/\text{day} \end{bmatrix} \qquad \textbf{S.21}$$

Figure 6(d)
$$\begin{bmatrix} A & B \\ C & D \end{bmatrix} = \begin{bmatrix} 0.459/\text{day} & \text{-}0.361/\text{day} \\ 0.391/\text{day} & 0.097/\text{day} \end{bmatrix} \qquad \textbf{S.22}$$

Even though Figure 6(*a*) and (*b*) illustrate qualitatively similar trajectories and velocity fields, the corresponding parameter values in equations S.19 and S.20 are different. In equation S.19, parameter *A* differs in value from parameter *B*, and parameter *C* differs from parameter *D*. This implies that both population *x* and population *y* have fitnesses that depend on the current composition of the population. However, in equation S.20, *A* equals *B*, and *C* equals *D*, which implies that the fitness of each cell type is independent of population composition. To demonstrate this point, substitute into equations 3.1 and 3.2 the parameter values from equation S.20 and note that the fractions p_x and p_y sum to unity. Thus, we obtain

$$\frac{\mathrm{d}x}{\mathrm{d}t} = \left(-0.2p_x - 0.2p_y\right)x = -0.2\left(p_x + p_y\right)x = -0.2x \qquad \textbf{S.23}$$

$$\frac{\mathrm{d}y}{\mathrm{d}t} = \left(0.2p_x + 0.2p_y\right)y = 0.2\left(p_x + p_y\right)y = 0.2y \qquad \textbf{S.24}$$

a differential equation describing population *x* expanding with growth coefficient -0.2/day (negative “expansion” actually corresponds to population decay), independent of population composition, and a differential equation describing population *y* expanding with growth coefficient 0.2/day, also independent of population composition.

This example shows that it is sometimes possible for EGT-based equations to fit population dynamics even when cell-cell interactions are not an important effect in an experimental system. In these cases, we cannot rely on a failed validation to alert us to the possibility of adequately interpreting data using simple models in which the cell-cell interactions so crucial to EGT are omitted. Figure 6(*a*) and (*b*) are similar, indicating that biological systems in which cell-cell interactions can be neglected can generate dynamics that qualitatively resemble the dynamics from some systems that do involve cell-cell interactions. A cursory inspection of a velocity field might not immediately reveal when an interaction-independent model of growth would be adequate. Taken together, this example shows that the dynamics of populations growing without cell-cell interactions (1) can pass the validation method described in subsection 3.2 and (2) can be described by phase portraits that look like phase portraits from systems in

which cell-cell interactions *do* contribute. Since validation and visual inspection of phase portraits might not alert us to the opportunity of using simple models in which cell-cell interactions are neglected, careful scrutiny of parameter values is required.

6.3. Three-population examples

Figure 6(*e*)
$$\begin{bmatrix} A_{xx} & A_{xy} & A_{xz} \\ A_{yx} & A_{yy} & A_{yz} \\ A_{zx} & A_{zy} & A_{zz} \end{bmatrix} = \begin{bmatrix} 0.689/\text{day} & 0.084/\text{day} & 0.152/\text{day} \\ 0.748/\text{day} & 0.229/\text{day} & 0.826/\text{day} \\ 0.450/\text{day} & 0.913/\text{day} & 0.538/\text{day} \end{bmatrix} \quad \textbf{S.25}$$

Figure 6(*f*)
$$\begin{bmatrix} A_{xx} & A_{xy} & A_{xz} \\ A_{yx} & A_{yy} & A_{yz} \\ A_{zx} & A_{zy} & A_{zz} \end{bmatrix} = \begin{bmatrix} 0.550/\text{day} & 0.208/\text{day} & 0.230/\text{day} \\ 0.622/\text{day} & 0.301/\text{day} & 0.844/\text{day} \\ 0.587/\text{day} & 0.471/\text{day} & 0.195/\text{day} \end{bmatrix} \quad \textbf{S.26}$$

Figure 6(*g*)
$$\begin{bmatrix} A_{xx} & A_{xy} & A_{xz} \\ A_{yx} & A_{yy} & A_{yz} \\ A_{zx} & A_{zy} & A_{zz} \end{bmatrix} = \begin{bmatrix} 0.4/\text{day} & 0.1/\text{day} & 0.2/\text{day} \\ 0.8/\text{day} & 0.2/\text{day} & 0.4/\text{day} \\ 1.2/\text{day} & 0.3/\text{day} & 0.6/\text{day} \end{bmatrix} \quad \textbf{S.27}$$

Figure 6(*h*)
$$\begin{bmatrix} A_{xx} & A_{xy} & A_{xz} \\ A_{yx} & A_{yy} & A_{yz} \\ A_{zx} & A_{zy} & A_{zz} \end{bmatrix} = \begin{bmatrix} 0.2/\text{day} & 0.2/\text{day} & 0.2/\text{day} \\ 0.4/\text{day} & 0.4/\text{day} & 0.4/\text{day} \\ 0.6/\text{day} & 0.6/\text{day} & 0.6/\text{day} \end{bmatrix} \quad \textbf{S.28}$$

Figure 6(*g*) and (*h*) illustrate visually similar velocity fields and trajectories, but the parameter values used to generate these simplex plots are different, as shown in equations S.27 and S.28. The line of reasoning presented in the previous subsection applies to these plots and parameter values. Figure 6(*h*) illustrates that an EGT-based model and equations can accommodate population dynamics data from an experimental system that does not necessarily involve cell-cell interactions. Additionally, the dynamics in such a system can qualitatively resemble dynamics obtained from a system that does contain cell-cell interactions, as demonstrated by the similarities between (*g*) and (*h*). Thus, direct scrutiny of the

parameter values, like those in equations S.27 and S.28, can be necessary for determining whether independent-growth models would adequately describe a particular experimental system.

Supplemental reference

[1] Cannings C, Edwards AWF 1968 Natural selection and the de Finetti diagram *Ann. Hum. Genet., Lond.* **31** 421-428

Supplemental figures

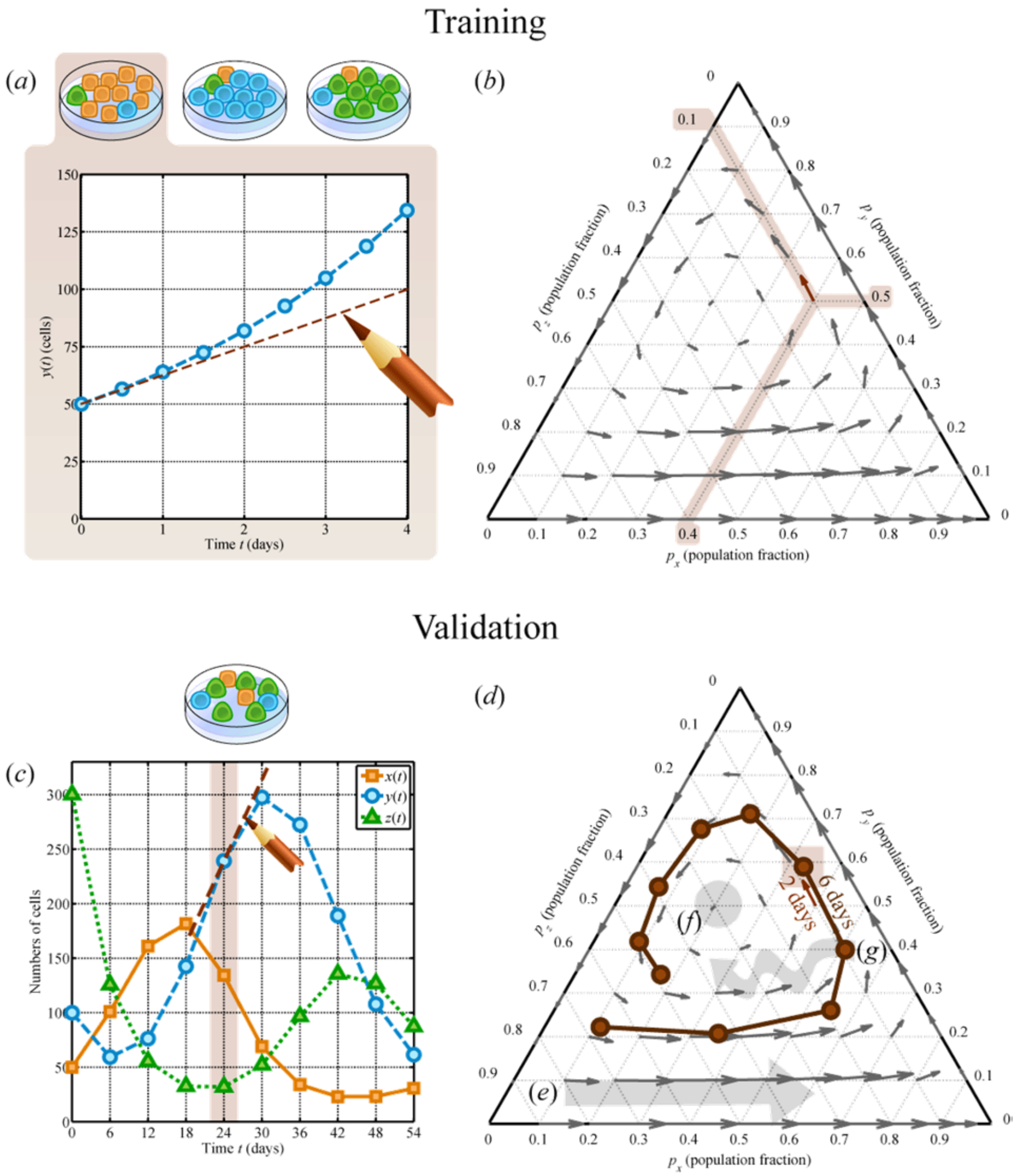

Figure 5. Training and validation of population dynamics equations for an ecology consisting of three populations. (*a*) Population *y* (round, blue cells) in a co-culture initially rich in cells of type *x* (square, yellow). The slope in (*a*) and eight additional population vs. time plots determine parameters in replicator equations represented by (*b*) a field of arrows on a simplex. (*c*) A separate dataset not used for parameter training is used (*d*) to validate the simplex. Examples of population compositions at which to initialize additional experiments are indicated at (*e*), (*f*), and (*g*).

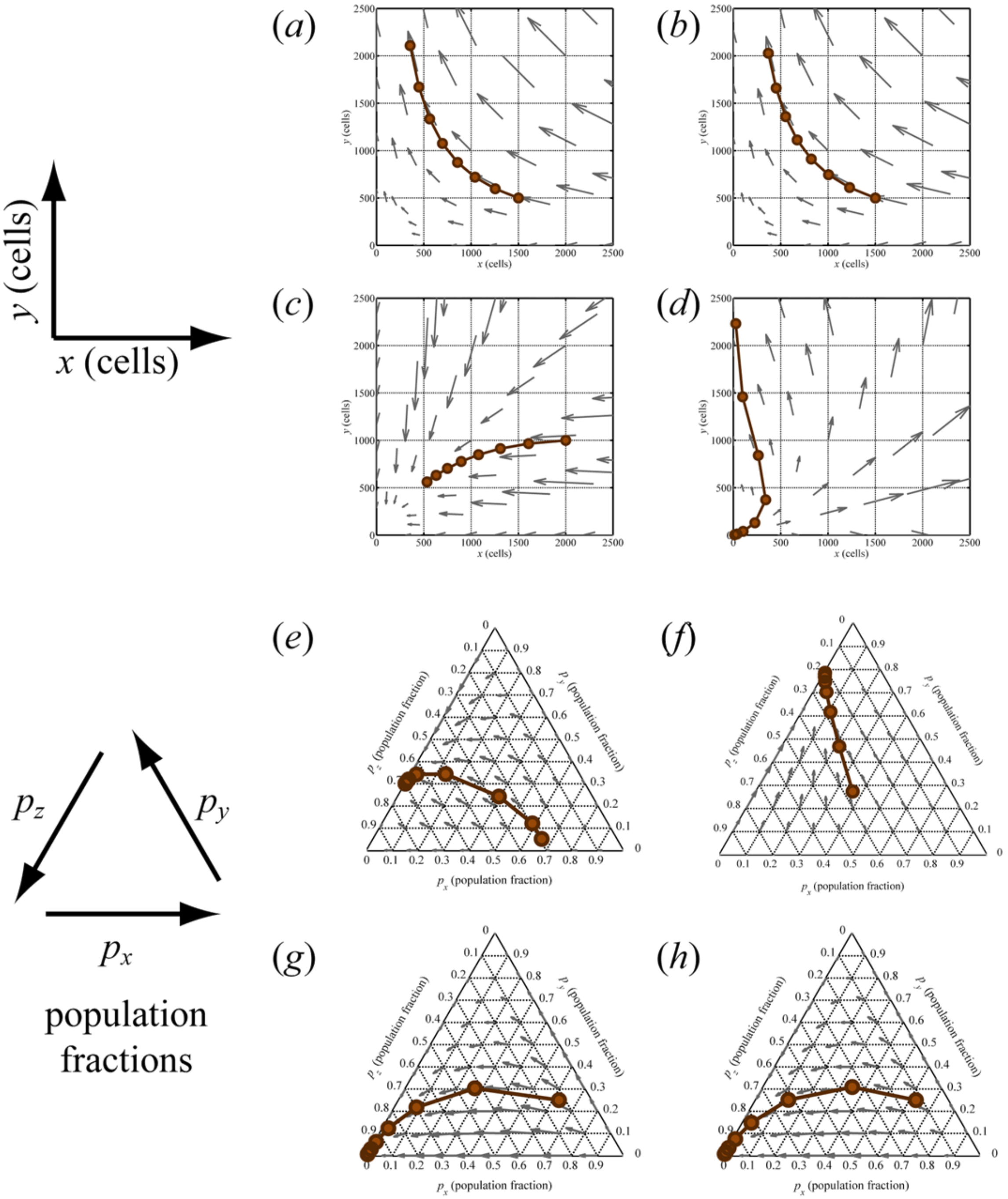

Figure 6. Examples of qualitative variety of trajectories that can be produced by replicator dynamics equations in (*a*)-(*d*) ecologies consisting of two populations and (*e*)-(*h*) ecologies consisting of three populations. Parameters can be found in a separate section of the supplemental materials. Even though panel (*a*) resembles (*b*) and (*g*) qualitatively resembles (*h*), these four panels actually correspond to different parameter sets.
